# Randomness of Eigenstates of Many-Body Quantum Systems

**DOI:** 10.3390/e21030227

**Published:** 2019-02-27

**Authors:** Li-Zhen Sun, Qingmiao Nie, Haibin Li

**Affiliations:** Department of Applied Physics, Zhejiang University of Technology, Hangzhou 310023, China

**Keywords:** randomness, quantum chaos, modulus fidelity

## Abstract

The emergence of random eigenstates of quantum many-body systems in integrable-chaos transitions is the underlying mechanism of thermalization for these quantum systems. We use fidelity and modulus fidelity to measure the randomness of eigenstates in quantum many-body systems. Analytic results of modulus fidelity between random vectors are obtained to be a judge for the degree of randomness. Unlike fidelity, which just refers to a kind of criterion of necessity, modulus fidelity can measure the degree of randomness in eigenstates of a one-dimension (1D) hard-core boson system and identifies the integrable-chaos transition in this system.

## 1. Introduction

Thermalization is a basic assumption in equilibrium statistical physics, although in general it is approached in real material. Then, in theoretical aspects, understanding the mechanism of thermalization in quantum many-body systems from microscopic quantum dynamic is a long debated problem and has seen renewed interests because of experimental progress in ultra-cold atom gases [1,2,3,4,5].

Random matrix theory (RMT) deals with the statistical properties of ensemble of matrices composed of random element which can model complex quantum many-body systems [6,7]. It is shown that the level spacing distribution of a random matrix obeys the Winger–Dyson distribution and the eigenstates of the random matrix are random vectors because the components of eigenstates satisfy a Gaussian distribution. The application of RMT to thermalization of a quantum many-body system leads to finding the key role of quantum chaos [8,9,10,11,12,13,14]. Eigenstate thermalization hypothesis (ETH) [9,10,11] has been conjectured so that in generic quantum many-body systems, thermalization occurs at individual energy eigenstates, which means that the expectation value of an few-body observable on one eigenstate equals to its microcanonical ensemble average. It has been verified numerically in a wide variety of quantum many-body systems in which the integrability is sufficiently destroyed [15,16,17,18,19], but it certainly does not hold in systems that are integrable or near integrable [15,16,17,18,19,20].

The breaking of integrability is related to the emergence of chaos. In quantum system, chaos refers to some specific properties of spectra, eigenstates, and dynamics [7,8,21]. Initial studies showed such characters in a kind of quantum systems which is chaotic in the classical limit [21]. Later, it was found that the same properties are intrinsic even in the quantum systems without classical counterparts. The statistical property of spectra is well described by the distribution of level spacing which changes from a Possion distribution to Winger–Dyson distribution in the transition from integrable to chaos [7]. Energy eigenstates should provide more information because of their own complicated structures which determine the expectation value of an observable. It is shown that when entering into chaos, the structure of eigenstates changes remarkably [7,8,21]. This scenario can be illustrated in the mean-field basis [22,23]. Commonly, a quantum many-body system described by Hamiltonian *H* can be separated into two parts as $H=H_{0}+H^{\prime }$, where $H_{0}$ is the Hamiltonian of noninteracting particles and $H^{\prime }$ is the interaction between particles, playing the role of perturbation. The mean-field basis is built by the eigenstates of $H_{0}$. When $H^{\prime }$ is zero, the system is integrable and thermalization is absent. Whereas when the perturbation is turned on, the integrability will be broken. The original unperturbed basis states are coupled with each other and form new eigenstates. However, as the interaction between particles is usually short-ranged, only neighbour unperturbed states are coupled directly and they will make up a subspace, corresponding to an energy shell [24,25]. At small perturbation, the number of unperturbed basis states contributing to one new eigenstate is small, meaning still a localized eigenstate. When the perturbation becomes large, more and more unperturbed states are coupled and new eigenstates can spread broadly in subspace and fulfill the shell. In ergodic cases [26,27], the expansion coefficients of new eigenstates in the mean-field basis satisfy a Gaussian distribution and these eigenstates are called random eigenstates or chaotic eigenstate [28]. Such a transition can be identified by a strength function which gives the distribution of square expansion coefficients in the mean-field basis [22]. In Ref. [29], the entanglement entropy of random vectors was obtained. Recently, entanglement entropy of eigenstates in quantum many-body fermion and boson system are investigated [30,31] to show their randomness by comparing with analytic result of random vectors.

ETH indicates that the expectation value of an observable on one enenrgy eigenstate is close to its neighbors in generic quantum many-body systems. Therefore, from above discussion, we can deduce that it is the random structure of energy eigenstates that leads to the closeness of expectation value of observable. In this paper, we will study how randomness emergences in the integrable-chaos transition in quantum many-body systems. For this purpose, two quantities, fidelity and modulus fidelity, are used. The rest of paper is organized as following. In Section 2, we will introduce the concept of fidelity and modulus fidelity and show analytic results of modulus fidelity between two random vectors. In Section 3, by using fidelity and modulus fidelity, we will investigate the closeness between eigenstates of a quantum many-body system and random vector to show the randomness in eigenstate structure. In Section 4 we will draw our final conclusion.

## 2. Fidelity and Modulus Fidelity between Two Random Vectors

Fidelity is a quantity to measure the closeness of two quantum states, including mixed state and pure state, in quantum mechanics and quantum information theory [32]. If just consider two pure quantum states denoted by |Φ〉 and |Ψ〉, fidelity is defined as quantity F=|〈Φ|Ψ〉|. When these two states are orthogonal, F=0, indicating that they are totally different. If two states are the same, F=1. In general, 0≤F≤1. Let’s consider two random vectors also denoted by |Φ〉 and |Ψ〉 in same Hilbert space with dimension *D* and a basis of this space is denoted by |n〉, then |Φ〉 and |Ψ〉 can be written as $\Phi =\sum_{n=1}^{D}\alpha_{n}|n\rangle$ and $\Psi =\sum_{n=1}^{D}\beta_{n}|n\rangle$, where $\alpha_{n}$ and $\beta_{n}$ are expansion coefficients. Without losing generality, let $\alpha_{n}$ and $\beta_{n}$ be real. Being the components of random vector, $\alpha_{n}$ and $\beta_{n}$ satisfy Gaussian distribution respectively. Moreover, they satisfy the norm condition for a quantum state, i.e., $\sum_{n=1}^{D}{\left|\alpha_{n}\right|}^{2}=1$, $\sum_{n=1}^{D}{\left|\beta_{n}\right|}^{2}=1$. The fidelity between them is $F=|\sum_{n=1}^{D}\alpha_{n}^{*}\times \beta_{n}|$ which is the absolute value of the mean of new random variable $\alpha_{n}^{*}\times \beta_{n}$. When *D* is infinite, it should be zero.

If the eigenstates of a quantum system, according to the principle of quantum mechanics, are orthogonal to each other, then the fidelity between any pair of them is strictly zero no matter what kind the quantum system is or what phase it occupies. It is trivial to study the closeness between eigenstates of quantum many-body systems by using fidelity. However, we can deal with fidelity between eigenstates of quantum many-body system and random vectors to find out whether these eigenstates are orthogonal to random vectors, which can give a necessary condition to judge whether a quantum state is random vector.

Moreover, we can use a modified fidelity, modulus fidelity, to measure the closeness between eigenstates of quantum many-body system and random vectors. Let’s still consider two states, |Φ〉 and |Ψ〉 in same Hilbert space with expansion coefficients $\alpha_{n}$ and $\beta_{n}$ respectively. In quantum mechanics, their modulus squared $|\alpha_{n}\left(\beta_{n}\right){|}^{2}$ mean the probability of finding the system in state |n〉. By taking the modulus of expansion coefficients of two states and substituting them into the definition of fidelity, modulus fidelity is defined as [33]

(1) $$M=\sum_{n=1}^{D}|\alpha_{n}\left|\right|\beta_{n}|.$$

In general, 0≤M≤1 as well as fidelity. For example, let’s consider two states of a two-level quantum system, $|\psi_{1}\rangle =\frac{1}{\sqrt{2}}(|1\rangle +|0\rangle$ and $|\psi_{2}\rangle =\frac{1}{\sqrt{2}}(|1\rangle -|0\rangle$, where |1〉 and |0〉 are the eigenstates of an observable $\hat{O}$ with eigenvalues, $o_{1}$ and $o_{2}$. The fidelity between them is F=0, meaning that they are orthogonal. If doing the measurement of observable $\hat{O}$, one can find that the probability of finding system at two eigenstate bases, |1〉 and |0〉, or getting the same measurement result, $o_{1}$ and $o_{2}$, are both 1/2. This result can be identified by the modulus fidelity between these two states because M=1. However, it is evident that, unlike fidelity, modulus fidelity is dependent on the choice of basis.

As mentioned in the introduction, random matrix theory describes the random structure of eigenstate of quantum many-body systems. We consider modulus fidelity between the eigenstates of a random matrix. Assuming two random vectors denoted by |Φ〉 and |Ψ〉 and expanded in a given orthonormal basis, the expansion coefficients are $\alpha_{n}$ and $\beta_{n}$ respectively. We first consider the case that $\alpha_{n}$ and $\beta_{n}$ are real and satisfy Gaussian distribution with zero mean and unit variance, then the probability density function is $f\left(x\right)=\frac{1}{\sqrt{2\pi }}exp\left(-x^{2}/2\right))$. If the dimension *D* is infinite, we can replace $\alpha_{n}$ and $\beta_{n}$ by continuous variables *x* and *y* and calculate modulus fidelity by integral,

(2) $$M=\int_{0}^{\infty }\int_{0}^{\infty }|x||y|J\left(x,y\right)dxdy,$$

where J(x,y) is the joint distribution function for x,y and equals to the product of probability function of *x* and *y*, i.e., J(x,y)=f(x)∗f(y), since *x* and *y* are independent random variables. Then we can obtain $M=\frac{2}{\pi }$.

Next, we consider the case that the expansion coefficients of two random vectors are complex and they are still denoted by $\alpha_{n}$ and $\beta_{n}$. Their real parts and imaginary parts satisfy Gaussian distribution with zero mean and unit variance respectively. It can be deduced that the fidelity between two such complex random vectors is also zero if the dimension *D* becomes infinite. To calculate the modulus fidelity, we take the modulus of expansion coefficients, $|\alpha_{n}|$ and $|\beta_{n}|$, and find them to satisfy the Rayleigh distribution with probability function $r\left(x\right)=xexp\left(-x^{2}/2\right)$, rather than the Gaussian distribution. Then, the modulus fidelity between these two complex random vectors can also be obtained by integral in a continuous limit,

(3) $$M=\int_{0}^{\infty }\int_{0}^{\infty }|x||y|J\left(x,y\right)dxdy,$$

where J(x,y)=r(x)∗r(y) is joint probability function. Under the normalization condition, we can obtain M=π/4.

## 3. The Numerical Results of a Quantum Many-Body System

In this section, we will use the quantities of fidelity and modulus fidelity to investigate the randomness in eigenstates of one-dimension(1D) hard-core boson model with Hamiltonian,

(4) $$\begin{array}{c} H=\sum_{i=1}^{N}\{-t\left({\hat{b}}_{i}^{†}b_{i+1}+H.c.\right)-t^{\prime }\left({\hat{b}}_{i}^{†}b_{i+2}+H.c.\right)+V{\hat{n}}_{i}{\hat{n}}_{i+1}+V^{\prime }{\hat{n}}_{i}{\hat{n}}_{i+2}\}, \end{array}$$

where *t* and $t^{\prime }$ are the nearest-neighbor hopping and the next-nearest-neighbor hopping, *V* and $V^{\prime }$ are the nearest-neighbor and the next-nearest-neighbor interaction respectively. Throughout this paper, *t* and *V* are set to be unity, t=V=1, and $t^{\prime }$ and $V^{\prime }$ are set to be equal, $t^{\prime }=V^{\prime }$, and ${\hat{n}}_{i}={\hat{b}}_{i}^{†}{\hat{b}}_{i}$ is a density operator. As is well known, this model is integrable when $t^{\prime }=V^{\prime }=0.0$. In this case, the Hamiltonian can be mapped onto non-interacting fermion system through the Jordan–Wigner transformation [34]. When $t^{\prime }=V^{\prime }\neq 0.0$, the system is non-integrable and comes into the chaos region when $t^{\prime },v^{\prime }$ increase. It has been verified that the thermalization can be achieved in this system and the underlying mechanism is ETH when system is far from integrable [15,16,19].

Using the exact diagonalization method, we can get all eigenstates and eigenvalues of the present system. Under the period boundary condition, the system preserves translational symmetry, by which the Hilbert space of the Hamiltonian can be decomposed into different independent subspaces with different total momentum *k*. We can diagonalize each subspace. Throughout this paper, without losing generality, we illustrate our finding by using the results of the eigenstates in subspace with momentum k=1, which can also avoid a parity symmetry conserved in subspace with k=0. The expansion coefficients of energy eigenstates are complex.

**Figure 1:**
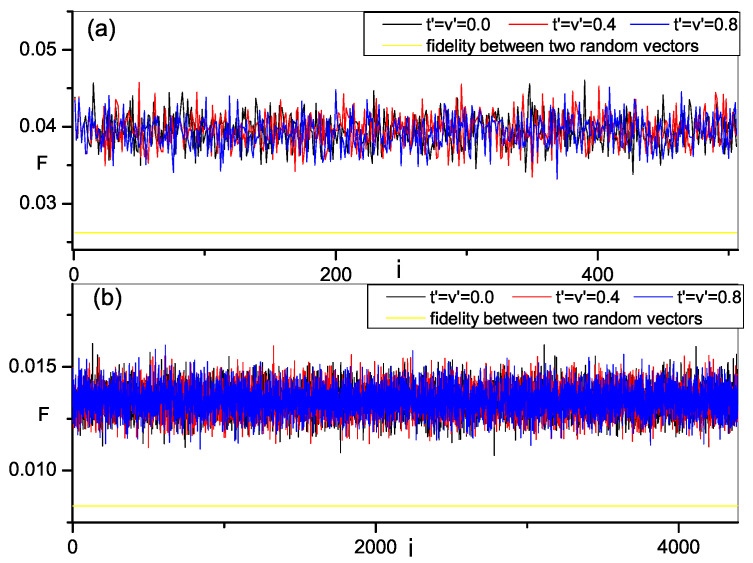
Fidelity between eigenstates of 1D hard-core boson system and random vector versus the index of eigenstate *i*, the results are averaged over 100 random vectors. The yellow solid line is the fidelity between two random vectors with same dimension. The dimension of Hilbert space *D*: (**a**) D=506; (**b**) D=4389.

As discussed previously, the fidelity between two random vectors is zero when dimension is infinite. However, if the dimension is finite, it is not zero and can be obtained by a numerical method. We also compute the fidelity between eigenstates of present model and complex random vector with the increase of the strength of integrability breaking perturbation $t^{\prime },v^{\prime }$ and plot the results in Figure 1. Different dimensions of the Hilbert space, representing different system size of the model, are considered. The results are obtained by averaging over an ensemble of random vectors. We note that the fidelity between every eigenstate and random vectors is finite and a little larger than that between two random vectors with same dimension, in both integrable region and chaos region. In this sense, one might say that all eigenstates of present model are close to random vectors even at integrability. However, as is well known, at leat most energy eigenstates of integrable quantum many-body systems are not random vectors [7]. So, from this result, we can only conclude that the eigenstates of the present model at integrability are close to being orthogonal to the random vector, but cannot judge whether the eigenstates are random vectors. In addition, we also find that the fidelity between different eigenstates and random vectors are also different, showing a distribution of fidelity with a fluctuation when corresponding eigenenergy varies. With the increase of strength of perturbations, the profile of the distribution of fidelity is kept such that it is still a little bigger than that between two random vectors, and their value regions are almost the same as that at integrability. We cannot find the difference in behavior of fidelity in these two cases and identify the integrable-chaos transition. However, it is not contradictory because the orthogonality to the random vectors is only a necessary condition to judge whether a quantum state is random vectors.

**Figure 2:**
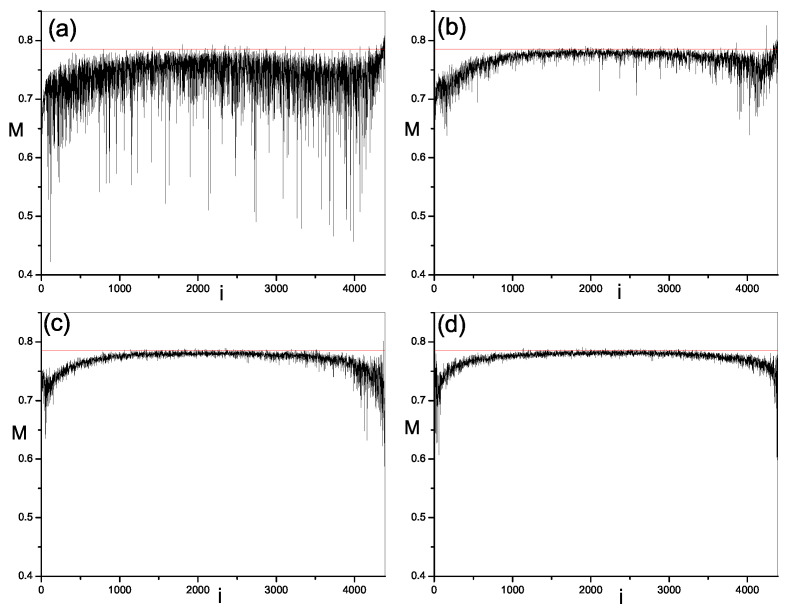
Modulus fidelity between eigenstates in 1D hard-core boson model in momentum basis and random vectors versus the index of eigenstate *i*. The dimension of space under consideration is D=4389. The results are averaged over 100 random vectors. The red solid line is the analytic result of modulus fidelity between two random vectors. (**a**) $t^{\prime }=v^{\prime }=0.0$; (**b**) $t^{\prime }=v^{\prime }=0.1$; (**c**) $t^{\prime }=v^{\prime }=0.4$; (**d**) $t^{\prime }=v^{\prime }=0.8$.

**Figure 3:**
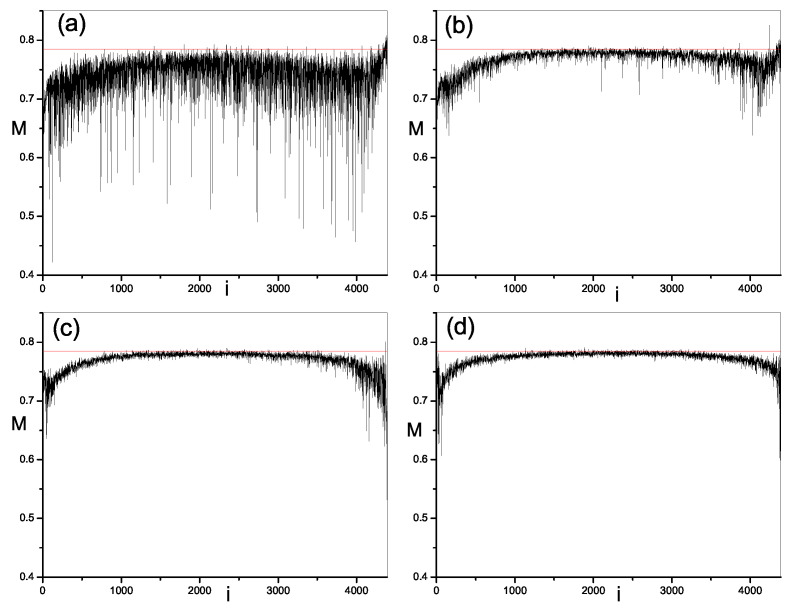
Modulus fidelity between eigenstates in 1D hard-core boson model in site basis and random vectors versus the index of eigenstate *i*. The dimension of space is D=4389. The results are averaged over 100 random vectors. The red solid line is the analytic result of modulus fidelity between two random vectors. (**a**) $t^{\prime }=v^{\prime }=0.0$; (**b**) $t^{\prime }=v^{\prime }=0.1$; (**c**) $t^{\prime }=v^{\prime }=0.4$; (**d**) $t^{\prime }=v^{\prime }=0.8$.

**Figure 4:**
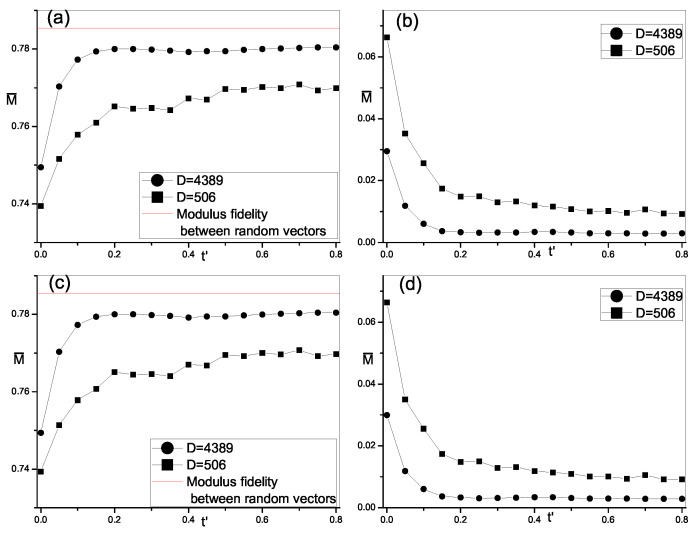
Average M(i), i.e., modulus fidelity between eigenstates of 1D hard-core model and random vectors versus the next-nearest-neighbor hopping $t^{\prime }$ in momentum basis (**a**) and site basis (**c**). The variance of M(i) versus $t^{\prime }$ in momentum basis (**b**) and site basis (**d**).

**Figure 5:**
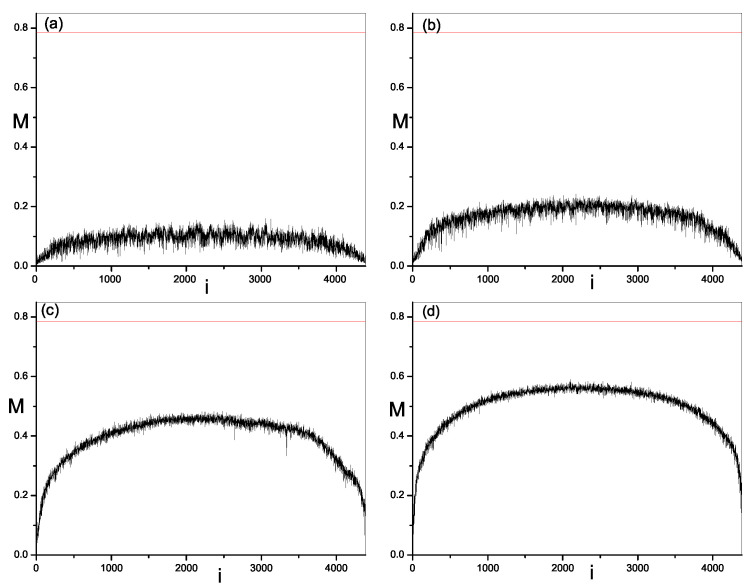
Modulus fidelity between eigenstates in 1D hard-core boson model in mean-field basis and random vectors. The dimension of space is D=4389. The results are averaged over 100 random vectors. The red solid line is the analytic result of modulus fidelity between two random vectors. (**a**) $t^{\prime }=v^{\prime }=0.05$; (**b**) $t^{\prime }=v^{\prime }=0.1$; (**c**) $t^{\prime }=v^{\prime }=0.4$; (**d**) $t^{\prime }=v^{\prime }=0.8$.

Next, we study the modulus fidelity between eigenstates of the present model and random vectors. By measuring the closeness between one eigenstate and random vectors, we can show the randomness in this eigenstate. As mentioned above, modulus fidelity is dependent on the basis. We investigate its behavior in three kinds of bases; momentum basis, site basis and mean-filed basis. The results are plotted in Figure 2, Figure 3, Figure 4 and Figure 5 respectively. As shown in Section 2, we have found the analytic result of modulus fidelity between two complex random vectors and denote it here by $M_{0}$, i.e., $M_{0}=\pi /4$, which is also plotted in Figure 2, Figure 3 and Figure 4 for comparison. The modulus fidelity between one eigenstate and random vector is written as M(i), where *i* denotes the index of eigenstate. For clarity, the difference between M(i) and $M_{0}$ is denoted by δM(i), i.e., $\delta M\left(i\right)=M\left(i\right)-M_{0}$.

In Figure 2, Figure 3, Figure 4 and Figure 5, we find the distribution of modulus fidelity M(i) is changing with the increase of strength of perturbation. However, the scenarios are complex in these three bases. In momentum basis and site basis, M(i) show similar behaviors. In both integrable and non-integrable cases, each value of M(i) is larger than 0.5 in the sense that all eigenstates of quantum system are close to the random vector, rather than far away. However, the degree of closeness between eigenstates and random vectors is much different in the integrable region and chaos region. When the perturbations are zero, i.e., the system is integrable, we notice that the absolute values of δM(i) of some eigenstates are small, indicating that these eigensates are very close to random vectors, while the absolute difference δM(i) of other eigenstates are bigger, which indicates that these eigenstates are not so close to random vectors. These two kinds of eigenstates distribute alternately in a spectrum which makes a large fluctuation of M(i) and δM at integrability. When the perturbations are turned on and become large, the integrability is broken and modulus fidelity M(i) of most eigenstates, in the middle of spectrum, increase and become close to $M_{0}$. More remarkably, the fluctuation of M(i) and δM is reduced dramatically. More and more eigenstates become close to random vectors. In addition, we notice that the situation in two edges of the spectrum is different from above results in middle of spectrum. With the increase of perturbation $t^{\prime },v^{\prime }$, although M(i) of most eigenstates in the edge are also increasing, but they will not be very close to $M_{0}$, meaning less random than the ones in middle of spectrum. On the other hand, the fluctuation of M(i) in the edges is also large even in the chaos region.

Considering the significant of fluctuation reduction, we calculate average and standard variance of modulus fidelity M(i) in momentum basis and site basis. Due to the boundary effect, the average is over the eigenstates in the middle of spectrum, which is 40% of all eigenstates. The results are plotted in Figure 4. In both momentum basis and site bases, for different system sizes, we notice that the average value of M(i) increases with the increase of perturbations. There exists a crossover in the increase of average modulus fidelity. It first increases quickly when perturbations increase from zero until a certain value, beyond which it increases slowly and tends to be saturated. In latter cases, average modulus fidelity becomes more close to $M_{0}$. When the dimension of space becomes large, this crossover becomes more evident and the saturated average value of modulus fidelity also increases. On the other hand, with the increase of perturbations, the fluctuation of modulus fidelity reduces remarkably in both momentum basis and site basis. At integrability, variance is large, indicating large fluctuation. It reduces quickly, first when perturbations increase. After that, it also experiences a crossover into a slow reduction and approaches a saturated value which is close to zero, corresponding to the smooth distribution of modulus fidelity in Figure 2 and Figure 3. Moreover, we can note that when the system size becomes large, the fluctuation of modulus fidelity in both integrable and chaos reduce accordingly. It is clear that the changes in both the average and variance show same crossover, which indicates the emergence of randomness in the structure of energy eigenstate and the transition from integrable to chaos. The value of perturbation at which crossover occurs is about $t^{\prime }=v^{\prime }\approx 0.2$ and becomes smaller when the system size increase as seen in Figure 4. It is regarded as a critical value for the appearing of transition and believed to approach to zero in the thermodynamic limit [16,19,22].

In the mean-field basis, the scenario is totally different from the momentum basis and site basis. In both integrable and chaos region, M(i) of all eigenstates are much smaller than those in momentum basis and site basis. At integrability, M(i) and δM(i) are very small and tend to be zero when the system size becomes large, due to the particularity of basis and we haven’t plotted them in the figure. When the perturbations are turned on and increase, as seen in Figure 5, M(i) of all eigenstates also increase, but keep close to zero, rather than π/4, even the perturbations are larger as $t^{\prime }=v^{\prime }=0.4$. In this sense, it seems that in the mean-field basis the eigenstates are not close to random vectors and the randomness of eigenstates is not revealed, while at same parameters eigenstates in momentum basis and site basis show sufficient randomness. However, if recalling the building of mean-field basis, one can understand such a contradictory result. As mentioned in the introduction, due to the short-range of interaction, only the neighbor eigenstate are coupled, so the spread of eigenstates is mainly in a eigenenergy shell, not in all space, with the increase of the integrability breaking perturbation. That is to say, the random structure of eigenstates in mean-field basis is local. Then using modulus fidelity to measure eigenstate in mean-field basis is to project local random structure onto global random structure, which leads to small modulus fidelity. Even so, as the modulus fidelity M(i) keeps increasing with the increase of perturbation and the magnitude of increase is much larger than that in momentum basis and site basis, it is more evident to see the spread of eigenstates and the production of randomness in eigenstate in mean-field basis than other two bases.

## 4. Discussion and Summary

In this paper, we investigated the randomness of eigenstates of a quantum many-body system by using the quantities, fidelity and modulus fidelity. We found that fidelity between each eigenstate and random vector is finite and a little larger than that between two random vectors. Especially, such scenario will not change with the increase of the integrability breaking perturbations. So, fidelity just provides some information about the necessary condition of random structure and cannot distinguish the chaos from integrability.

To identify the randomness in eigenstates of a quantum many-body system in the integrable–chaos transition, we used modulus fidelity to measure the closeness between eigenstates of quantum many-body system and random vectors. For this purpose, we first studied the modulus fidelity between two random vectors and found analytic results which will be a criterion to judge how random a quantum state is. Numerical results of a one-dimensional hard-core boson system show that modulus fidelity can be used as a quantity to distinguish the integrable (or near integrable) region from chaotic region, although it is dependent on basis. In the momentum basis and site basis, when the system is chaotic, the modulus fidelity between most eigenstates in the middle of spectrum of present quantum system and random vectors are surely very close to that between two random vectors. Furthermore, the reduction of the fluctuation in modulus fidelity is a more significant phenomena in the transition from integrable to chaos. In the integrable region, the fluctuation of modulus fidelity is prominent which makes eigenstates show different structure from their neighbors. In the chaotic region, the fluctuation is reduced remarkably such that most eigenstates, mainly in the middle of spectrum, are close to random vectors. Then they become similar with neighbours, which has been confirmed by our previous work on the closeness of eigenstates in the same quantum many-body system [33]. That is the underlying reason for the validity of the eigenstate thermalization hypothesis, that in chaotic quantum systems the expectation value of an observable on one eigenstate is close to that of the neighbour eigenstate. In the mean-field basis, we found that the random structure of eigenstates is local, which makes the modulus fidelity smaller, seemingly showing less randomness. In fact, based on the result of fidelity and modulus fidelity in momentum basis and site basis, we can find that even at integrability, the energy eigenstates of quantum many-body system also show randomness. But in the building of the mean-field basis, the unperturbed energy eigenstates become the bases and the randomness in them is hidden, which makes the modulus fidelity between eigenstates and random vectors close to zero. The next-near-neighbor interaction, causing the destruction of integrability, couples the neighbor unperturbed states and leads to the randomness in eigenstates under perturbation, which is distinct in the mean-field basis.
